# De novo mutation of emopamil binding protein (*EBP*) gene in a girl with Conradi‐Hünermann‐Happle syndrome

**DOI:** 10.1002/ccr3.2213

**Published:** 2019-06-28

**Authors:** Ana Soler‐Cardona, Oliver Brandau, Franco Laccone, Adrian Tanew, Sonja Radakovic

**Affiliations:** ^1^ Department of Dermatology Medical University of Vienna Vienna Austria; ^2^ Institute of Medical Genetics Medical University of Vienna Vienna Austria; ^3^ Center for Human Genetics Mannheim Mannheim Germany; ^4^ Private Dermatological Practice Vienna Austria

**Keywords:** alopecia, cataract, genodermatosis, pigmentary disorders

## Abstract

Conradi‐Hünermann‐Happle syndrome is a rare X‐linked dominant syndrome affecting the skin, skeletal system, and eyes. Here, we report on a female patient with a de novo heterozygous missense mutation c.301C>T (p.Trp101Arg) of the EMP (emopamil binding protein) gene.

## 1 INTRODUCTION

Ichthyoses are a large group of skin diseases characterized by scaling of the skin with or without erythroderma.^1^ A common denominator in the pathology of ichthyoses is a defective epidermal barrier. Integrity of the epidermal barrier is mediated by an extracellular lamellar bilayer surrounding the cornified cells in the stratum corneum. The lipid compartment of the lamellar bilayer is composed of ceramides, cholesterol, and free fatty acids.^2^ Inborn errors in cholesterol metabolism lead to increased toxic cholesterol metabolites and cholesterol deficiency in the cell membranes with consequent barrier function alteration. The Conradi‐Hünermann‐Happle (CHH) syndrome (X‐linked dominant chondrodysplasia punctate, CDPX2 [Online Mendelian Inheritance in Man 302960]) is an X‐linked dominant ichthyosiform condition that results from genetic mutations in the distal part of the cholesterol pathway.^3^ As of December 2018, 90 mutations have been reported in the HGMD Professional (version 2018.3) database, a registry of gene lesions in human inherited disease. We here describe the case of a female Caucasian infant who was found to have CHH syndrome with a previously unidentified heterozygous mutation in the emopamil binding protein (*EBP*) gene.

## 2 CASE PRESENTATION

**Figure 1:**
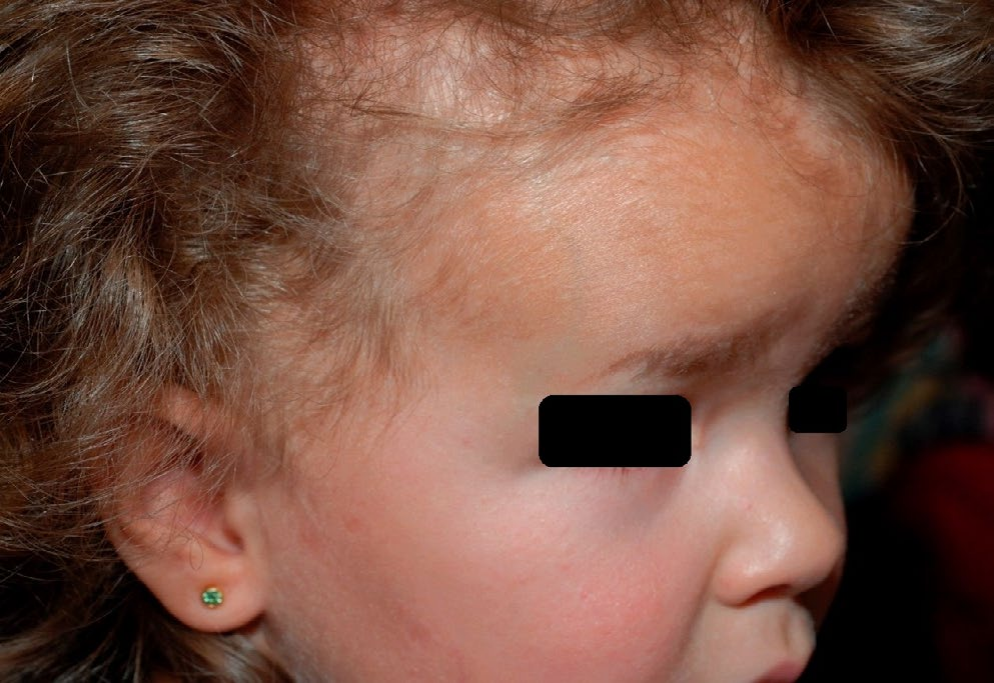
Facial dysmorphism with a flat nasal bridge, prominent frontal bossing, and maxillary hypoplasia

A two‐and‐a‐half‐year‐old female infant was referred to us from the pediatric department with a suspected diagnosis of incontinentia pigmenti and tinea capitis. The child was born full term by Caesarean section after an uneventful pregnancy. At birth, a deformation of the lower jaw and a generalized, scaly erythema of the skin were present. Erythroderma resolved during the first year of life and was replaced by hypopigmentation (Figure 1). A previous physical examination had not revealed any other abnormal findings except a diagnosis of cataracts. An X‐ray examination had been rejected by parents. The girl was the only child of no consanguineous parents, and the family history was negative for genetic skin diseases.

**Figure 2:**
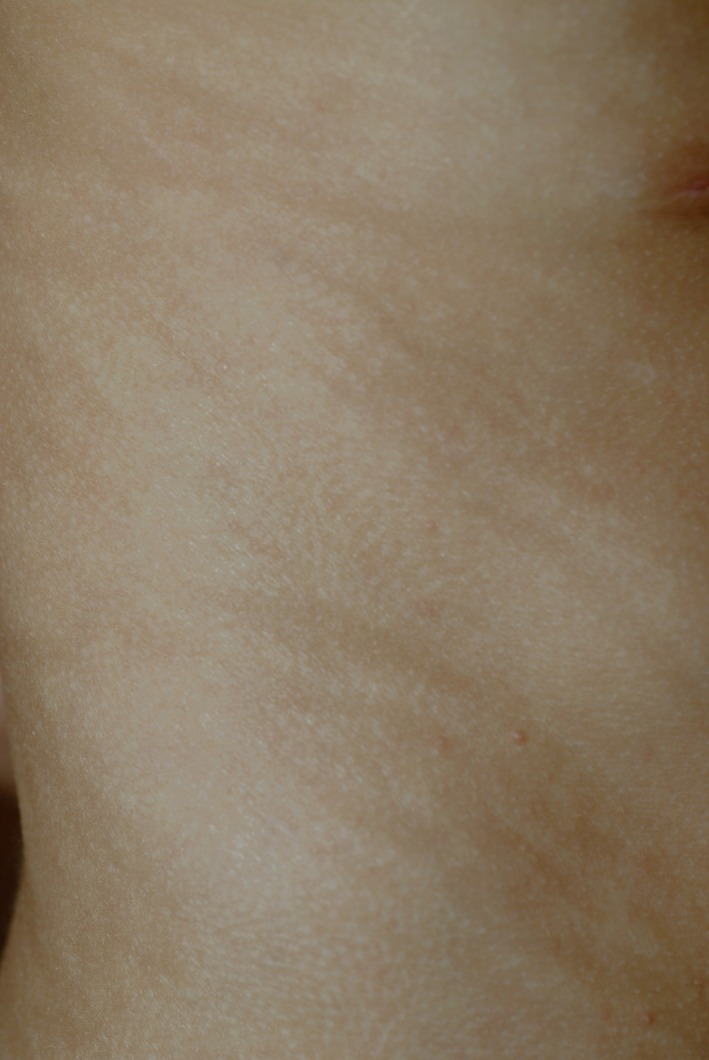
Hypopigmentation along the lines of Blaschko on the left side of the trunk

**Figure 3:**
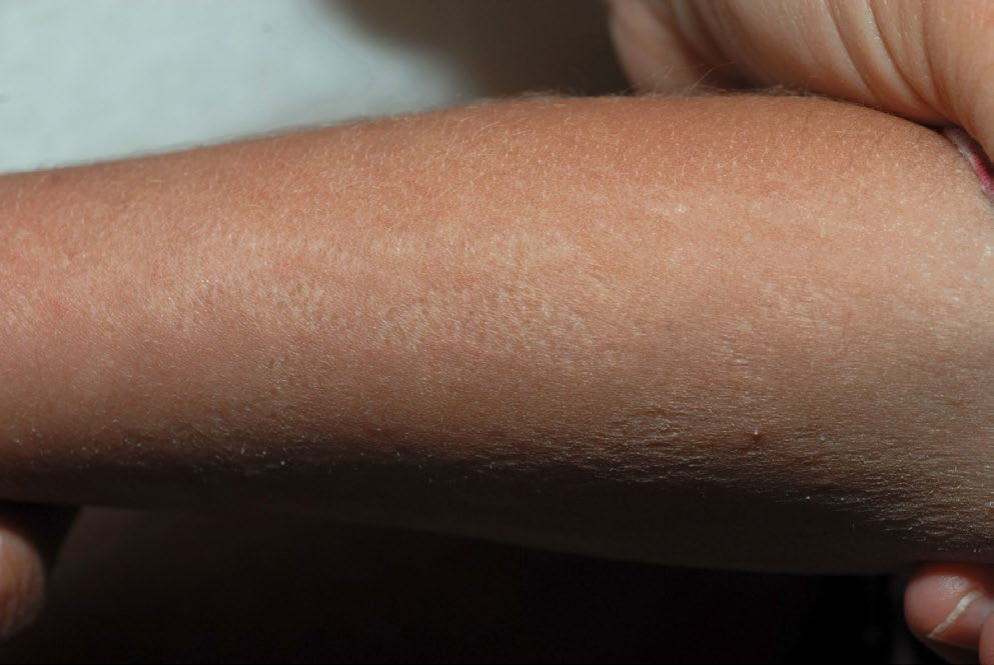
Follicular atrophoderma of the forearms

**Figure 4:**
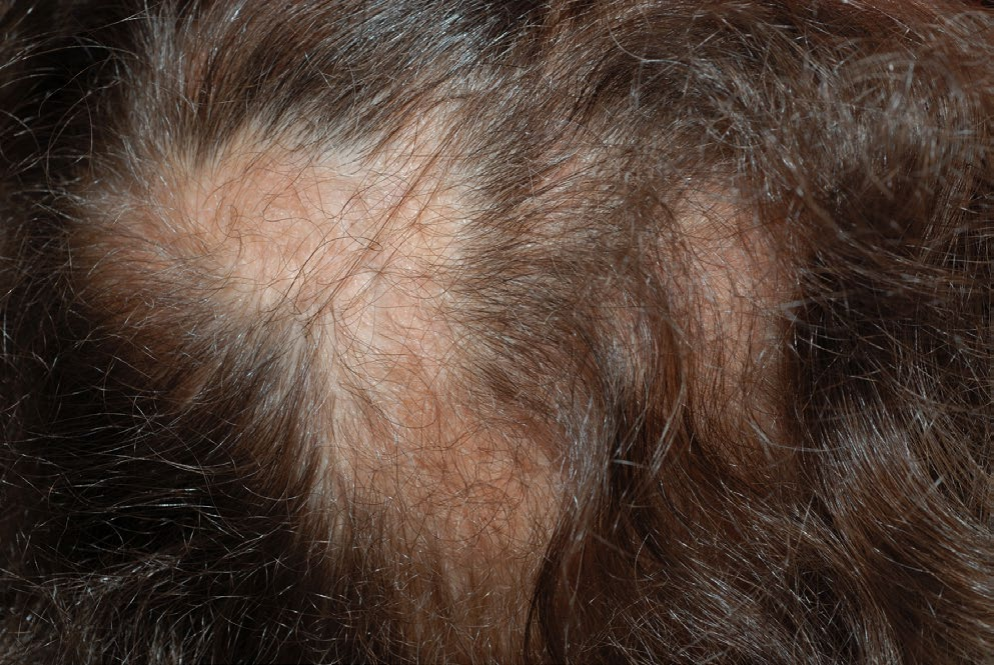
Patchy scarring alopecia

On dermatological examination, there was facial dysmorphism with a flat nasal bridge, prominent frontal bossing, and a maxillary hypoplasia (Figure 1). The skin showed a generalized hypopigmentation distributed along the lines of Blaschko (Figure 2), a mild ichthyosis of the entire body, atrophoderma with dilated follicular openings, and ice pick scars that were most pronounced on the extremities (Figure 3) and a patchy scarring alopecia (Figure 4). The nails and teeth were normal.

A genetic analysis ruled out deletion of exons 4‐10 of the NEMO gene and thus did not support the initial differential diagnosis of incontinentia pigmenti. The diagnosis of CHH syndrome was confirmed by subsequent genetic analysis that identified a heterozygous base exchange at base pair 301 of the coding sequence of the *EBP* gene (c.301T>C) in the exon 2 resulting in a missense mutation (p.Trp101Arg) of the corresponding protein. This mutation has so far not been reported in a patient or in control databases (1000 genomes) and computer programs. The in silico evaluation of the variant predicted it as pathogenic (MutationTaster, Polyphen‐HumVar, SIFT). Parental genetic testing showed a wild‐type sequence in both of them indicating a de novo origin of the mutation.

## 3 DISCUSSION

Conradi‐Hünermann‐Happle is a rare X‐linked dominant ichthyosiform condition affecting approximately 1:400 000 births.^4^ Mutations in the *EBP* gene at Xp11.22‐23 result in deficiency of 3β‐hydroxysteroid‐Δ8,Δ7‐isomerase enzyme (also called EBP protein) in the distal pathway of cholesterol biosynthesis (squalene to cholesterol).^5^ The enzyme deficiency leads to accumulation of 8(9)‐cholestenol and 8‐dehydrocholesterol which can be used as biochemical markers. Diagnosis is based on clinical suspicion in case of ichthyosis following the lines of Blaschko in a female patient and confirmation by mutation analysis of the EPB gene.^3^ Detection of the above‐mentioned biomarkers in plasma, scales from skin lesions, fibroblasts, or lymphoblasts aid in the diagnosis.^3^ Since CHH is an X‐linked dominant inherited disease, the disease is normally lethal for males and females are affected in 95% of the cases. However, cases of male patients with Klinefelter syndrome or normal karyotype (and somatic mosaicism) have been identified.^3^ In males, it is important to differentiate between CHH due to a mosaic phenotype and the recently described nonmosaic X‐linked recessive MEND syndrome (*m*ale *E*BP disorder with *n*eurological *d*efects) since prognosis and clinical outcome are different.^6^ In contrast to CHH, the hypomorphic mutations occurring in MEND syndrome are associated with serious neurological defects (cerebellar hypoplasia, hydrocephalus, hypoplasia of corpus callosum, and Dandy‐Walker malformation) leading to seizures, developmental delay, decreased muscle tone (hypotonia), or death shortly after birth.^6^

So far, 90 *EBP* gene mutations have been detected including *missense* and *nonsense* mutations specially affecting the exons 2 and 4 as well as deletions and insertions.^7^ The vast majority of mutations affect the exons, but also splice‐site mutations have been described.^3^ A heterozygous missense mutation c.139T>C has been associated with developmental delay and behavioral difficulties in four male family members.^8^ So far, no genotype‐phenotype correlation has been established although some authors claim a more severe phenotype among patients with *nonsense* mutations.^9^

Conradi‐Hünermann‐Happle is an uncommon genetic disorder of the skin, skeletal system, and eyes with heterogeneous clinical manifestation.^10^ The skin is affected in 95% of the cases and presents with congenital ichthyosiform erythroderma, atrophoderma with dilated follicular openings, and hypopigmentation along the lines of Blaschko. Occasionally, a linear or whorled hyperkeratosis may be seen; however, vesicles are always absent. The scalp involvement includes patchy scarring alopecia with or without lusterless and twisted hair. Onychoschisis may be present. Skeletal involvement comprises anomalies of the face (flattened nose bridge and frontal bossing), malformation of limbs (joint dysfunction, hexadactyly, and shortening of long bones), anomalies of the vertebral column leading to scoliosis, and stippling of the epiphyses of long bones (chondrodysplasia punctata).^3^, ^10^ Bone defects start soon after birth with punctate calcifications resulting from abnormal calcium deposition during endochondral bone formation. These changes are found predominantly in the epiphyses of the long bones and usually disappear during adulthood; however, the short bones and trachea can also be affected.^3^ Up to 67% of the patients have cataracts (uni‐ or bilateral) that are already present at birth or develop early in life. Occasionally, other eye findings such as microphthalmia, microcornea, glaucoma, or atrophy of the optic nerve have been reported. Anomalies of the CNS, ears, kidneys, and heart have also been described.^10^

Management of CHH is symptomatic and multidisciplinary and includes orthopedic care and cataract extraction. Skin treatment is restricted to the use of emollients and keratolytics.

In summary, we here report on a female child with a typical presentation of CHH in whom subsequent genetic testing confirmed the clinical diagnosis and identified a hitherto unreported mutation in the exon 2 of the EBP gene resulting in a missense mutation. Our case illustrates that due to its rarity, variable clinical presentation and similar distribution pattern of the skin lesions CHH might be easily confused with and misdiagnosed as incontinentia pigmenti (IP). However, several clinical features point toward a diagnosis of CHH. As opposed to incontinentia pigmenti, CHH does not evolve through different stages and never presents with vesicles. Follicular atrophoderma with ice pick‐like depressions of the skin and scarring alopecia are further distinguishing signs of CHH. In addition, CHH is associated with characteristic facial dysmorphism and other skeletal features that are not found in IP. Awareness of this rare genodermatosis and genetic analysis will allow establishing an early correct diagnosis.

## CONFLICT OF INTEREST

None declared.

## AUTHOR CONTRIBUTION

AS‐C: participated in generating the data for the study, participated in gathering the data for the study, wrote the original draft of the paper, and approved the final version of this paper. OB: participated in generating the data for the study, participated in gathering the data for the study, did the genetic analysis, and participated in writing the paper. FL: participated in generating the data for the study, participated in gathering the data for the study, did the genetic analysis, participated in writing the paper, and approved the final version of this paper. AT: participated in generating the data for the study, participated in gathering the data for the study, participated in writing the paper, and approved the final version of this paper. SR: participated in generating the data for the study, participated in gathering the data for the study, participated in writing the paper, reviewed the pertinent raw data on which the results and conclusions of this study are based, and approved the final version of this paper.
